# Where Is the Extended Phenotype in the Wild? The Community Composition of Arthropods on Mature Oak Trees Does Not Depend on the Oak Genotype

**DOI:** 10.1371/journal.pone.0115733

**Published:** 2015-01-30

**Authors:** Martin M. Gossner, Martin Brändle, Roland Brandl, Johannes Bail, Jörg Müller, Lars Opgenoorth

**Affiliations:** 1 Terrestrial Ecology Research Group, Department of Ecology and Ecosystem Management, Technische Universität München, Hans-Carl-von-Carlowitz-Platz 2, 85354 Freising, Germany; 2 University of Marburg, Department of Ecology, Karl-von-Frisch Strasse 8, D-35043 Marburg, Germany; 3 University of Erlangen-Nuremberg, 91058 Erlangen, Germany; 4 Bavarian Forest National Park, Freyunger Str. 2, 94481 Grafenau, Germany; Umeå Plant Science Centre, Umeå University, SWEDEN

## Abstract

Through a series of common garden experiments, it has been shown that heritable phenotypic differences between individual trees can affect arthropod communities. However, field studies under heterogeneous environmental conditions remain rare. In the present study, we investigated the genetic constitution of 121 mature oak host trees at different trophic levels from 10 sites across Bavaria, southern Germany and their associated insect communities. A total of 23,576 individuals representing 395 species of beetles and true bugs were evaluated. In particular, we determined whether the composition of arthropod communities is related to the oak genotype and whether the strength of the relationships decreases from lower to higher trophic levels, such as for phytophagous, xylophagous, zoophagous, and mycetophagous species. The genetic differentiation of oaks was assessed using eight microsatellite markers. We found no significant influence of the oak genotype on neither the full beetle and true bug community nor on any of the analyzed trophic guilds. In contrast, the community composition of the insects was highly related to the space and climate, such that the community similarity decreased with increases in spatial distance and climatic differences. The relationship with space and climate was much stronger in beetles than in true bugs, particularly in mycetophagous species. Our results suggest that spatial processes override the genetic effects of the host plant in structuring arthropod communities on oak trees. Because we used neutral markers, we cannot exclude the possibility that trait-specific markers may reveal a genetic imprint of the foundation tree species on the composition of the arthropod community. However, based on the strength of the spatial patterns in our data set, we assume that genetic differences among oaks are less important in the structuring of arthropod communities. Future whole-genome studies are required to draw a final conclusion.

## Introduction

Intraspecific genetic diversity is an important driver of ecological processes, such as primary productivity, population recovery from disturbance, interspecific competition, community structuring, and fluxes of energy and nutrients [1]. In particular, the impact of intraspecific genetic variation on the community structure of associated organisms has been viewed in terms of the extended phenotype [1]. Since the classic studies conducted by Whitham et al. [2], a number of empirical studies have provided evidence supporting the significant cascading effects of genetic variation within foundation species on the composition and diversity of associated communities (community phenotype) and ecosystem processes (ecosystem phenotype) [3–7].

The majority of these studies used common garden approaches to exclude confounding environmental and spatial factors [6]. Model species, such as *Populus* and *Salix*, are commonly used because they exhibit high levels of intraspecific genotypic variation, including a large number of hybrid species. More importantly, these species are known to display strong phenotypic differences between genotypes, such as the composition of secondary compounds and phenology [8,9]. Although these approaches support the extended phenotype concept, it remains unclear whether the effects of the extended phenotype are also relevant in natural systems exposed to heterogeneous environmental conditions [1,10]. For a thorough discussion of these approaches and their pitfalls, see the report published by Tack and colleagues [11].

However, few studies have attempted to trace the effects of the extended phenotype in wild foundation tree populations. In temperate regions, Whitham and coworkers [12–14] used a wild and common garden cottonwood population and compared the respective extended phenotype effects on arthropod communities. Encouragingly, both systems showed similar results. Although neither the arthropod species richness nor abundance differed significantly among the cottonwood cross types, significant differences were found in the arthropod community composition. The studies conducted by Tack et al. [10,15] compared the effects of genotype on the arthropods associated with oaks both in common garden settings and wild populations. In particular, the objective of this study was to estimate the relative effects of the host plant genotype, environment, and genotype-environment interactions on the species richness of herbivores on *Quercus robur* across different scales. However, the population spatial effects, such as connectivity and spatial autocorrelation, were more important for the definition of species richness than the genetic makeup of the tree.

In this study, we extended the approach used by Tack et al. [10] in several ways. (1) The genetic diversity of *Quercus robur* is comparatively low, as was shown for the southern Finnish range by Mattila and coworkers [16] and Vakkari and coworkers [17]. Therefore, we studied mixed populations of the potentially hybridizing *Quercus petraea* and *Quercus robur* populations to increase the genetic diversity of our dataset. (2) Prior studies have suggested that the plant genotype is more likely to structure the arthropod community composition than the arthropod abundance or species richness [12]. Therefore, we focused on the similarity in species composition, which may be a more sensitive measure for detecting the extended phenotype effects in the wild. (3) The genetic composition of foundation species, such as the individual tree genotypes of *Populus angustifolia*, can affect higher trophic levels via cascading effects to herbivorous and carnivorous arthropods and insectivorous birds. This further extends to soil microbial communities with significant consequences on ecosystem processes [18]. Consequently, we included different trophic levels for true bugs (Hemiptera: Heteroptera) and beetles (Coleoptera) in our analyses.

We tested (1) whether the effects of the oak genotype on arthropod communities can be observed in the wild and (2) whether the impact decreased from lower to higher trophic levels. We sampled both the arthropod community and oak genetic data from a total of 121 trees across ten sites within Bavaria, southern Germany. We predict that (1) the arthropod community composition is related to the genetic composition and, to a lesser degree, to the spatial distance between oaks because species are hypothesized to cope better with the expected low environmental and climatic differences across Bavaria than with differences in the genetic and consequently the chemical composition of plant tissues; (2) the magnitude of the effect of the arthropod responses on the genetic differences between trees differ among trophic levels and decrease with increasing trophic level such that canopy dwelling leaf phytophages and xylophages are more highly affected than zoophages and mycetophages because they are more strongly related to the oak chemical composition; and (3) the leaf phytophage effects are stronger in chewers than suckers because chewers must cope with more secondary plant compounds than suckers such that they consume a greater range of different plant tissues with different chemical compounds.

## Material and Methods

### Ethics statement

Field work permits were issued by the responsible state environmental offices of Bavaria, including the regional administrative authorities of Lower, Upper and Middle Franconia, Lower and Upper Bavaria, and Swabia. All of the studied forests were state forests with the exception of Iphofen, in which the local forester granted us the corresponding rights for our research activities. No protected species were sampled.

### Study system and sites

**Fig 1:**
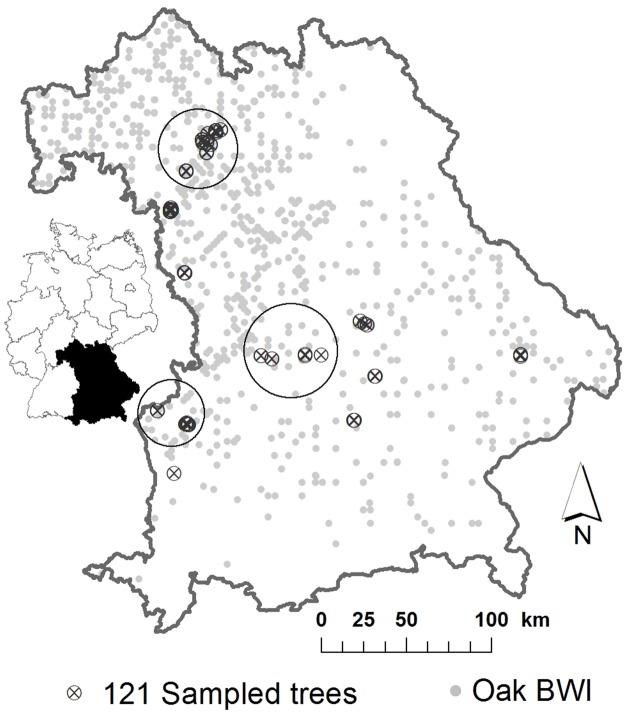
Location of studied oak trees. Distribution of the sampling sites of the 121 studied oak trees (black crossed circles) across 10 forest sites in Bavaria, Southern Germany. One site (“population”) was defined by a minimum distance of 20 km to the next site. Trees within a large circle were assigned to one “population”. The light grey circles indicate the occurrence of oaks in Bavaria according to the German forest inventory (BWI), a nationwide terrestrial forest inventory sampling procedure with permanent sampling points.

In this study, we analyzed the insect assemblages and genetic composition of 121 adult oak trees (age > 100 years) from 10 forest sites covering the entire spatial range of oaks across the German federal state of Bavaria (Fig. 1). We restricted our study to Bavaria to minimize the differences in regional species pools caused by geological and historical constraints, but we included different regions across Bavaria to cover all main oak woodland communities of Southern Germany, which differ greatly in terms of environmental conditions. The analyzed oak trees belong to two species, sessile oak (*Quercus petraea* (Matt.) Liebl.) and pedunculate oak (*Quercus robur* L.), which are closely related, often co-occur and sometimes interbreed. Both species are important in timber production and cover large parts of Europe [19]. Oaks are rich in insect species with a high number of specialists [20,21]. Brändle and Brandl [21] reported a total of 699 phytophagous insect and mite species on oaks in Germany, of which 252 are limited to feeding on this host genus.

### Genetic characterization of oak trees

We sampled leaves from all individual trees using either a shot gun or a crossbow. For genetic characterization of the oaks, a highly validated eight-plex microsatellite setup specifically developed for *Quercus petraea* and *Quercus robur* was used [22]. The genomic DNA from approximately 70 g of dried leaf material was extracted using the DNeasy Plant Kit (Qiagen, Hilden) according to the manufacturer’s instructions. Multiplex PCRs were performed according to the protocol developed by Guichoux et al. [22] and were scored by an external laboratory (Ecogenics, Switzerland). The genetic distances between the individual trees were calculated as Manhattan distances following the method described by Smouse and Peakall [23] using Genalex 6.4. Other distance measures, such as delta mu and R_ST_, were also calculated and produced similar results (data not shown). To delimitate *Quercus robur* and *Quercus petraea* and their hybrids, the microsatellite data were subjected to a structure analysis [24] by setting two clusters as prior (structure 2.3.1). Based on Guichoux and coworkers [22], assignment thresholds of 0–0.25 and 0.75–1 for purebreds and 0.25–0.75 for F1 hybrids were used.

### Characterization of true bug and beetle communities

The species richness and abundance of true bugs (Hemiptera: Heteroptera) (http://dx.doi.org/10.6084/m9.figshare.1272803) and beetles (Coleoptera) (http://dx.doi.org/10.6084/m9.figshare.1272802) were assessed using flight-interception traps (FITs) [25]. These were installed in the core of each tree crown (installation height 16–33 m, depending on the tree height). Sampling jars were filled with non-attractant 1.5% CuSO_4_–solution (for details see [26]). Each tree was sampled from mid-March through mid-October in one year during the period of 1996 to 2004. The traps were emptied monthly, and the arthropods were transferred to 70% ethanol in the field. Monthly samples from each trap were pooled for further analyses.

Determinations at the species level were performed by either one of the authors (MMG, Heteroptera) or by taxonomic specialists recruited for the project (Coleoptera). For subsequent analyses, species of sucking Heteroptera were mainly assigned to phytophagous and zoophagous species according to Wachmann et al. [27]. The nymphs of the former group of species feed on plants, whereas animals dominate the diets of the species in the latter groups. Chewing Coleoptera were assigned to phytophagous leaf chewer species and to xylophagous, mycetophagous and zoophagous species among saproxylics according to Koch [28,29]. Data for the other trophic guilds of beetles, such as the zoophagous non-saproxylics and saprophagous species, were not available. Details on the species classification criteria used are provided in S1 Table. All of the subsequent analyses were performed on the total dataset and the trophic guild subsets.

To estimate the compositional dissimilarity of the respective species compositions of true bugs and beetles, we calculated the Bray-Curtis dissimilarity and species turnover using the Simpson dissimilarity [30] (for an overview of different beta-diversity indices, please refer to [31]) based on the log-transformed data using the vegan 2.03 package within the R software [32].

### Climatic and spatial data

For the climatic characterization of the sites, we first calculated the values for the 19 BIOCLIM variables using the ‘biovars’ method of the R package dismo 0.9–1 (http://dx.doi.org/10.6084/m9.figshare.1272790). We then scaled the 19 BIOCLIM variables and conducted a Principal Component Analysis (PCA) using the ‘princomp’ function. In the subsequent analyses, we used the most important resulting principal components, i.e., those with an eigenvalue higher than the average eigenvalue. To reduce these principal components to a single matrix, we calculated a Euclidian distance matrix with the ‘vegdist’ function using the R package vegan 2.03. Similarly, the ‘vegdist’ function was used to generate a geographic distance matrix based on the x and y coordinates.

### Data analyses

Dissimilarity matrices of the true bug and beetle sets and subsets were correlated with the genetic distances, geographic distances, and climatic parameters by Mantel tests and partial Mantel tests using the R package vegan based on Pearson’s product correlations. The spatial autocorrelation of the oak genetic distances was analyzed as described by Smouse and Peakall [23,33–36] using Genalex 6.4. This method allows the inclusion of multivariate data, such as the combination of different loci in the analysis. The generated autocorrelation coefficient is closely related to Moran’s I and can be interpreted in the same manner. The same method was used to analyze the spatial autocorrelation in the community data of the true bugs and beetles. For the analysis, the above-mentioned dissimilarity matrices (true bugs and beetles) and distance matrix (oaks) were used. The analysis was performed with the following settings: 22 10-km-wide distance classes, each with 999 permutations and bootstrap replicates. The results of the autocorrelation analysis of the oak genetic data and the community data of true bugs and beetles are shown and summarized in a correlogram generated using Genalex.

## Results

### Genetic characterization of oaks

As expected in highly outcrossing tree species, the intra-population genetic diversity was high. In particular, of the 121 trees from 10 plots included in the analysis, all eight microsatellite loci analyzed were variable, with 15 (MsQ13), 16 (QrZAG20), 18 (QpZAG15), 19 (QrZAG112), 22 (QrZAG7 and QrZAG96), 23 (QpZAG110), and 34 (QrZAG11) size variants. Of these, 36 alleles belonged to *Q*. *petraea*, 53 belonged to *Q*. *robur* alone, and 80 of the 169 alleles occurred in both species. Of the 169 alleles, 39 were unique to one population. All of the populations had at least one private allele (4.9±1.6). Structural analysis with strict thresholds (0.25–0.75%) revealed that only three trees were assigned a hybrid F1 status. Of the ten populations, three and four were purely composed of *Quercus robur* and *Q*. *petraea*, respectively, whereas the remaining three populations contained both species (see S1 Fig.).

### Characterization of arthropod communities

In total, we sampled 5,943 individuals (mean ± SE: 49 ± 3 per tree) belonging to 76 true bug species (10 ± 0.3) and 17,633 beetles (146 ± 10) belonging to 319 species (29 ± 0.8) (both on 121 trees). Of the true bugs, 3,596 individuals (30 ± 3) belonging to 46 species (7 ± 0.3) were phytophagous, and 2,347 individuals (19 ± 1) belonging to 30 species (3 ± 0.2) were zoophagous. Of the beetles, 8,461 individuals (70 ± 6) belonging to 99 species (9 ± 0.4) were phytophagous, 2,626 individuals (22 ± 1) belonging to 151 species (10 ± 0.4) were xylophagous, 4,289 individuals (36 ± 3) belonging to 46 species (4 ± 0.2) were mycetophagous, and 2,257 individuals (19 ± 5) belonging to 75 species (5 ± 0.2) were zoophagous.

### Effect of genetic composition of foundation tree species, space and climate on true bug and beetle communities

**Fig 2:**
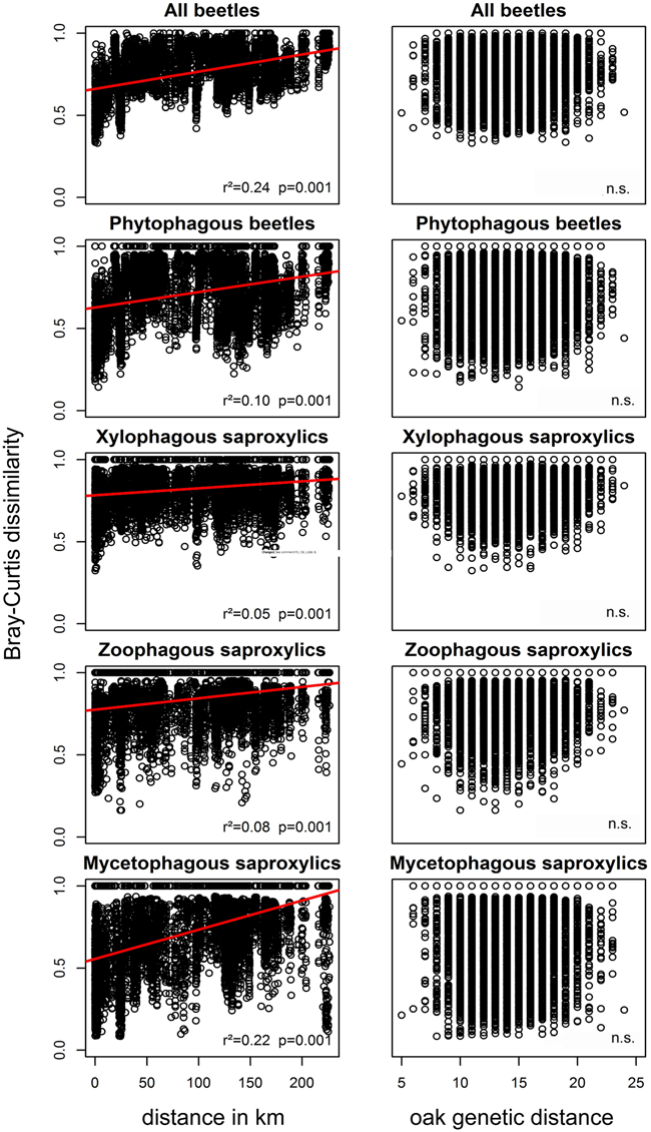
Beetle communities vs. geographic and genetic distance. Bray-Curtis dissimilarities of the beetle communities plotted against geographic distances (in km) and against genetic distances (Manhattan distances). The r² and p values correspond to the results from the respective Mantel tests. A regression line is plotted in red.

**Fig 3:**
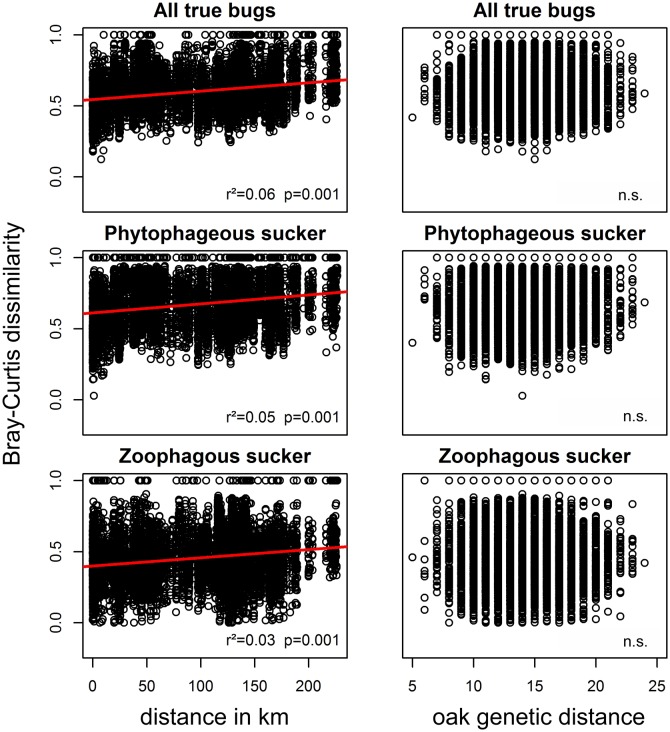
True bug communities vs. geographic and genetic distance. Bray-Curtis dissimilarities of the true bug communities plotted against geographic distances (in km) and against genetic distances (Manhattan distances). The r² and p values correspond to the results of respective Mantel tests.

The genetic composition of the trees did not affect the true bug and beetle assemblages, regardless of the trophic group (Table 1, Fig. 2, Fig. 3), even when we corrected for climate or space in multiple Mantel tests (Table 2). This finding was confirmed by analyzing the species turnover (Simpson dissimilarity) of beetles and true bugs (S2 and S3 Tables).

**Table 1:** Mantel test between the oak genotype, space, climate and arthropod assemblages.

Set1	Set2	r_M_	Significance
Beetles			
All beetles	OGD	0.045	
Phytophagous leaf chewer	OGD	0.023	
Xylophagous saproxylics	OGD	0.047	
Zoophagous saproxylics	OGD	0.038	
Mycetophagous saproxylics	OGD	0.049	
All beetles	Space	0.49	***
Phytophagous leaf chewer	Space	0.31	***
Xylophagous saproxylics	Space	0.23	***
Zoophagous saproxylics	Space	0.28	***
Mycetophagous saproxylics	Space	0.47	***
All beetles	Climate	0.55	***
Phytophagous leaf chewer	Climate	0.46	***
Xylophagous saproxylics	Climate	0.26	***
Zoophagous saproxylics	Climate	0.26	***
Mycetophagous saproxylics	Climate	0.36	***
True bugs			
All true bugs	OGD	0.047	
Phytophagous sucker	OGD	0.049	
Zoophagous sucker	OGD	0.021	
All true bugs	Space	0.25	***
Phytophagous sucker	Space	0.22	***
Zoophagous sucker	Space	0.18	***
All true bugs	Climate	0.40	***
Phytophagous sucker	Climate	0.32	***
Zoophagous sucker	Climate	0.32	***
Oaks, Space, Climate			
Oak genetic distances	Space	0.085	**
Oak genetic distances	Climate	0.065	
Climate PCA	Space	0.57	***

Relationship between pair-wise community composition estimates (in transformed Bray Curtis dissimilarity index of true bugs and beetles) and oak genetic distances (OGD), spatial distance (Space), climatic differences (Climate) and among the OGD, Space and Climate distance measures. The results of the Mantel test based on Pearson’s product-moment correlations are provided. Set1 and Set2 indicate the first and second matrix of each Mantel test, respectively. Climate refers to the main climatic components (see Material and Methods). The significance levels are as follows: *** p<0.001, ** p<0.01, and *p<0.05.

**Table 2:** Multiple Mantel test between the oak genotype, space, climate and arthropod assemblages.

Set1	Set2	Corrected for	r_M_	Significance
Beetles				
All beetles	OGD	Climate	0.0052	
Phytophagous leaf chewer	OGD	Climate	-0.012	
Xylophagous saproxylics	OGD	Climate	0.029	*
Zoophagous saproxylics	OGD	Climate	0.045	
Mycetophagous saproxylics	OGD	Climate	0.020	*
All Beetles	OGD	Space	0.0042	
Phytophagous leaf chewer	OGD	Space	-0.0040	
Xylophagous saproxylics	OGD	Space	0.028	
Zoophagous saproxylics	OGD	Space	0.041	
Mycetophagous saproxylics	OGD	Space	0.0029	
All beetles	Space	Climate	0.26	***
Phytophagous leaf chewer	Space	Climate	0.075	**
Xylophagous saproxylics	Space	Climate	0.10	**
Zoophagous saproxylics	Space	Climate	0.16	***
Mycophagous saproxylics	Space	Climate	0.35	***
All Beetles	Climate	Space	0.39	***
Phytophagous leaf chewer	Climate	Space	0.36	***
Xylophagous saproxylics	Climate	Space	0.16	***
Zoophagous leaf chewer	Climate	Space	0.13	***
Mycetophagous saproxylics	Climate	Space	0.13	**
True Bugs				
True bugs	OGD	Climate	0.019	
Phytophagous sucker	OGD	Climate	0.026	
Zoophagous sucker	OGD	Climate	-0.034	
True bugs	OGD	Space	0.027	
Phytophagous sucker	OGD	Space	0.031	
Zoophagous sucker	OGD	Space	-0.023	
True bugs	Space	Climate	0.035	
Phytophagous sucker	Space	Climate	0.055	*
Zoophagous sucker	Space	Climate	0.0062	
True bugs	Climate	Space	0.32	***
Phytophagous sucker	Climate	Space	0.24	***
Zoophagous sucker	Climate	Space	0.26	***
OGD	Climate	Space	0.031	
OGD	Space	Climate	0.053	*

Relationship between the pair-wise community composition estimates (in transformed Bray Curtis dissimilarity index of true bugs and beetles) and oak genetic distances (OGD), spatial distance (Space), climate differences (Climate) and among the OGD, Space and Climate distance measures. The results are the means of multiple Mantel tests based on Pearson’s product-moment correlations and are provided. Set1, Set2 and ‘corrected for’ indicate the first, second and third matrixes of each partial Mantel test. The significance levels are as follows: *** p<0.001, ** p<0.01, and *p<0.05.

**Fig 4:**
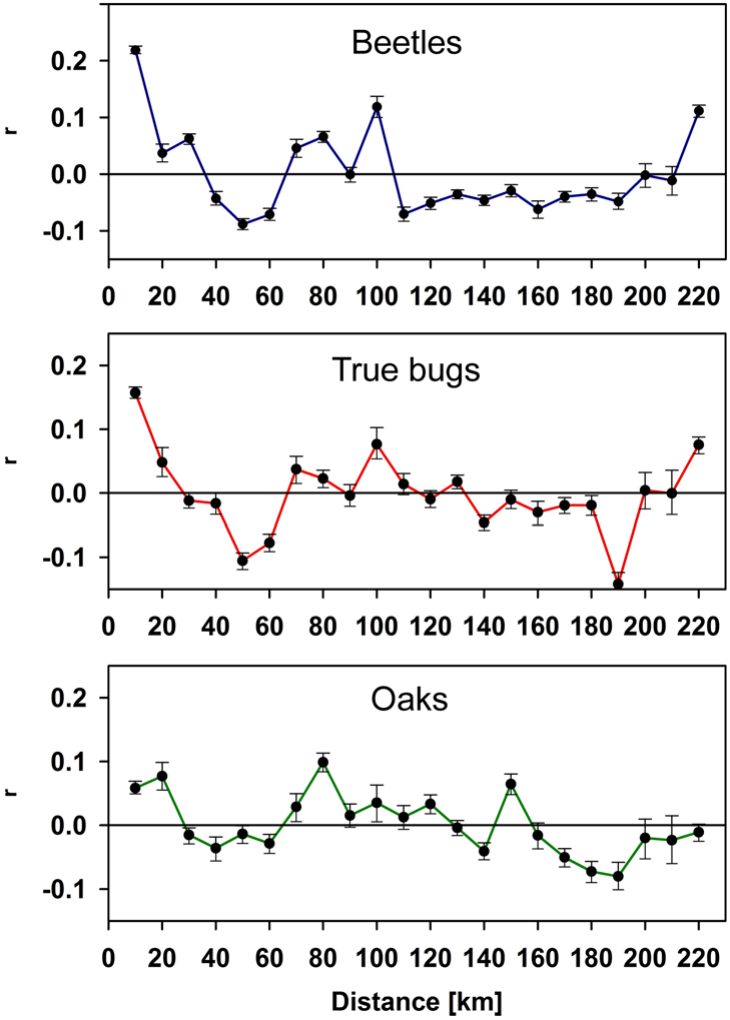
Community similarity of true bugs, beetles and oaks vs. geographic distance. Correlogram of the community similarity of beetles, true bugs, and oaks as a function of distance (in km). This correlogram is interpreted as Moran’s I.

The results of the PCA of the bioclim variables showed that the eigenvalues of the first four components contributed 94% to the correlations. Thus, for the subsequent analyses, a Euclidian distance matrix was built using these four components. The space and climate were highly correlated with each other (Table 2) and had significant effects on the arthropod community compositions. The effect was strongest in beetles, in which 30% and 24% of the variance was explained by the climate and space, respectively. In true bugs, only 16% and 6% of the variance was explained by the climate and space, respectively. Among the beetle guilds, the variance in the community assemblages was best explained for phytophages (21%, 10%), followed by mycetophages (13%, 22%), zoophages (7%, 8%) and xylophages (7%, 5%). In true bugs, climate explained 10% of the phytophagous and zoophagous assemblages, and space explained 5% and 3% of these assemblages, respectively. The effects of climate and space on the community composition were lower but still significant after correcting for the other in the partial Mantel test. The analysis of the spatial autocorrelation showed a significant positive spatial autocorrelation at spatial scales of up to 20 km for true bugs and oak genetics and up to 30 km for beetles (Fig. 4).

## Discussion

In our study, we assessed the community composition of true bugs and beetles on *Quercus robur* and *Quercus petraea* to quantify the effect of genetic variability in the foundation tree species on the arthropod community structure. Our data consistently suggested that the plant genotype has no significant effect on the structure of the beetle and true bug assemblages across the studied trophic levels. This finding is in contrast to the growing body of research in community genetics showing that the genetic diversity in host plants can significantly impact the structure of the associated assemblages (e.g., [6,37–46]). However, the relative importance of the host plant genetic background on the structure of the associated assemblages compared with other factors is largely unknown for most species. In many respects, this finding is indebted to the fact that community genetic approaches overlook natural populations and focus on common garden experiments [47]. In fact, the importance of scale in studies of community genetics has been emphasized by Tack et al. [11].

The community composition of the local fauna depends on a number of historical and recent factors. For example, historical factors comprise co-evolutionary histories of the host and associated organisms and more strictly biogeographical factors, such as glacial and postglacial imprints in the fauna [48]. Contemporary factors include current ecological interactions, current environmental conditions, and spatial variables that impact the dispersal ability of organisms [49,50]. The present study found that both climate and space significantly affect the community composition of beetles and true bugs. Space explained 24% of the beetle variance and 6% of the true bug variance, whereas climate explained 30% of the beetle variance and 16% of the true bug variance. These findings are comparable to those of other studies. For example, in a meta-analysis of environmental and spatial processes in ecological communities that included 158 studies, Cottenie [49] found that, on average and independent of the spatial scale (the studies varied greatly in spatial scale), 48% of the total variation in the community structure was explained by the combination of environmental and spatial variables, whereas 22% and 16% of the total variation was explained by purely environmental variables and by space alone, respectively. Although no studies on beetles and true bugs were included in this meta-analysis, the overall results are reasonably consistent with our estimates. However, the effect was found to vary substantially among the different guilds in the present study. For example, the effect of space and climate on xylophagous and zoophagous beetles in the present study was lower than of the all individually reported results from the 158 studies included in the meta-analysis. At present, we cannot suggest an explanation for this diverging pattern for independent guilds. Nevertheless, Soininen et al. [50] showed that the trophic position and dispersal type highly affect the distance-decay relationships; therefore, the differences in the analyzed groups may be a result of the combination of both of these variables. Space and climate were much better explanatory variables for individual guilds, such as mycetophagous saproxylics, than for others. Therefore, it appears that these variables act more strongly on some guilds than others, indicating that either the potential role of biotic processes on the structuring of these communities is smaller than anticipated or that other biotic drivers lie behind these patterns. This is consistent with the results of a study on beta-diversity in beech forests of Germany [51], in which significant differences in the variance were explained by space for various trophic groups of beetles, decreasing from mycetophages (16%) to herbivores (10%) and zoophages (10%) to xylophages (3%) (Gossner, unpublished data).

Our study does not identify why the respective guilds react differently to space and climate because the autecological knowledge on the different bug and beetle species remains limited. This is also true for the spatial dimension of the species ecologies, including the individual dispersal capabilities. Nevertheless, according to the correlogram, our results demonstrate a positive correlation between the arthropod communities at distances between 10 and 30 km for beetles and between 10 and 20 km for true bugs. No positive spatial autocorrelation was detected beyond 20–30 km. Therefore, unlike the findings reported by Tack et al. [10], who reported spatial structuring of herbivore communities on both local and regional scales, we only observed this on a local scale.

In both our study and the 158 studies included in the meta-analysis conducted by Cottenie [49], the largest part of the variance remained unexplained. Therefore, the following questions remain: (1) whether a genetic signal of the host plants explains a relevant portion of the variation in assemblages and (2) why so many of the recent community genetic studies show such an effect and our study did not. In a recent study, Tack and coworkers discussed a spatial bias introduced into many community genetics studies [11]. According to their argument, these studies included a genetic differentiation of foundation species at a large geographic scale while reducing the environmental settings to the single locality of the common garden experiments. Therefore, these studies excluded any spatial signal in the associated communities. As a consequence, the community genetic signal is disproportionally increased, and the studies produce unrealistic proportions of the explained variance. In our study, this spatial imbalance was not found because the scale of the oak genetic background is the same as that of the true bug and beetle assemblages.

Furthermore, the marker choice also has a significant impact. For example, in the study conducted by Wimp et al. [12], who used cottonwood as a model, 8% of the variance in the community composition was explained by the genetic composition of the host trees. However, it is known that differences in the genetic composition lead to marked differences in the chemical composition of plants. Naturally, this trait potentially affects the arthropod community composition. In our study, we used neutral markers, the linkage of which to potentially relevant genes is not known. Therefore, it is possible that specific genes encoding ecologically relevant traits influence the assemblages of associated organisms. This is a methodological dilemma because qualified background information is by definition not available for non-model species. To better assess the potential of neutral markers in the community genetics debate, it would be interesting to assess the genetic impact of model species on the associated organisms not only using the more powerful genomic resources that are currently available for the models but also using microsatellites to enable comparisons.

Another issue that may make comparisons between studies assessing the extended phenotypes difficult is that the different studies use different descriptors for the community effect. For example, the community composition has been shown to be relatively sensitive to host genetic signals. Furthermore, different trophic guilds should also show varying degrees of sensitivity depending on how closely they are linked to the host species. Numerous studies have reported that the extended phenotype effect on dependent herbivore communities cascades up to higher trophic levels (e.g., [52–57]). In contrast to these studies, we were unable to show a significant effect of genetic differences among the studied oaks on herbivores; therefore, it is not surprising that we also did not find a similar cascading effect to other trophic guilds.

Furthermore, the proportion of specialists vs. generalists or even tourists should have an impact on the potential strength of a host genetic signal in community assemblages. Robinson and coworkers reported the genetic variation in functional traits, particularly plant growth traits but also defensive chemical compounds, that influence arthropod communities in aspen. The majority of their morphospecies were reported to be specialists on the respective aspen species, and it is likely that co-evolutionary processes play an important role [45]. The same relatively high degree of specialization is true for sawflies on different *Salix* species, a model for which strong community genetic effects primarily associated with chemical defenses are documented [58]. Although the *Quercus* genus has a relatively large number of specialists with a co-evolutionary history [20], the specialization of the species pair *Q*. *robur* and *Q*. *petraea* is negligible compared with these examples [56,59]). For example, among the most specialized group of insects, namely the gall-inducers, preference for one of the studied oak species can hardly be found [60], and these species are thus combined in most evolutionary ecology studies [61].

In conclusion, the results of our study are consistent with the results reported by Tack and coworkers and show that the community composition in arthropods associated with *Quercus robur* and *Quercus petraea* has a clear spatial and climatic signal. This finding highlights the importance of considering regional species pools in all types of diversity studies and that the results must be discussed in the framework of distance decay and environmental constraints [62]. It seems plausible that the identification of extended phenotypes in the wild and the confirmation of their ecological relevance will be the exception rather than the rule. However, for a more informed discussion, additional studies across a range of taxonomic and functional groups and at different geographic scales are necessary, particularly in wild populations of non-model species. Furthermore, because specific genotype-phenotype links are usually not known for non-model species, it must first be determined in well-known model systems whether neutral markers are capable of detecting an extended phenotype.

## Supporting Information

S1 Fig Structure bar plot of the genetic membership proportions (*K* = 2). Each tree is represented by a vertical line divided in *K* colors.
(JPG)

S1 Table Occurrence table of beetles and true bugs. Each of the rows contains a single species. Each column represents one tree. The beetles are divided into mycetophagous, phytophagous, xylophagous, and zoophagous, and true bugs are divided into zoophagous and phytophagous.
(CSV)

S2 Table Mantel test between Oak genotype, space, climate and arthropod assemblages. Relationship between pair-wise community composition estimates (ln transformed Simpson dissimilarity index of true bugs and beetles) and oak genetic distances (OGD), spatial distance (Space), climate differences (Climate) and among the OGD, Space and Climate distance measures. The results of the Mantel test based on Pearson’s product-moment correlations are provided. Set1 indicates the first and Set2 indicates the second matrix of each Mantel test.
(DOCX)

S3 Table Multiple Mantel test between Oak genotype, space, climate and arthropod assemblages. Relationship between the pair-wise community composition estimates (ln transformed Simpson dissimilarity index of true bugs and beetles) and oak genetic distances (OGD), spatial distance (Space), climate differences (Climate) and among the OGD, Space and Climate distance measures. The results are the means of multiple Mantel tests based on Pearson’s product-moment correlations and are provided. Set1 indicates the first, Set2 indicates the second and ‘corrected for’ indicates the third matrix of each partial Mantel test.
(DOCX)

## References

[1] Hughes A, Inouye BD, Johnson MT, Underwood N, Vellend M (2008) Ecological consequences of genetic diversity. Ecology Letters 11 (6): 609–623. Available: WOS:000255552100008. 10.1111/j.1461-0248.2008.01179.x 18400018

[2] Whitham TG, Young WP, Martinsen GD, Gehring CA, Schweitzer JA et al (2003) Community and ecosystem genetics: A consequence of the extended phenotype RID A-8538–2009. Ecology 84 (3): 559–573.

[3] Whitham TG, Bailey JK, Schweitzer JA, Shuster SM, Bangert RK et al (2006) A framework for community and ecosystem genetics: from genes to ecosystems. Nat Rev Genet 7 (7): 510–523. Available: ISI:000238377200013. 16778835 10.1038/nrg1877

[4] Haloin JR, Strauss SY (2008) Interplay between Ecological Communities and Evolution Review of Feedbacks from Microevolutionary to Macroevolutionary Scales. Year in Evolutionary Biology 2008 1133: 87–125. Available: ISI:000260225000005. 10.1196/annals.1438.003 18559817

[5] Zytynska SE, Fay MF, Penney D, Preziosi RF (2011) Genetic variation in a tropical tree species influences the associated epiphytic plant and invertebrate communities in a complex forest ecosystem. Philosophical Transactions of the Royal Society B: Biological Sciences 366 (1569): 1329–1336. 10.1098/rstb.2010.0183 21444307 PMC3081567

[6] Whitham TG, Gehring CA, Lamit LJ, Wojtowicz T, Evans LM et al (2012) Community specificity: life and afterlife effects of genes. Trends Plant Sci 17 (5): 271–281. Available: 10.1016/j.tplants.2012.01.005 22322002

[7] Castagneyrol B, Lagache L, Giffard B, Kremer A, Jactel H (2012) Genetic diversity increases insect herbivory on oak saplings. PLoS ONE 7 (8): e44247 10.1371/journal.pone.0044247 22937168 PMC3429418

[8] Hochwender CG, Fritz RS (2004) Plant genetic differences influence herbivore community structure: evidence from a hybrid willow system. Oecologia 138 (4): 547–557. Available: ISI:000220365700007. 14727172 10.1007/s00442-003-1472-4

[9] Bailey JK, Schweitzer JA, Ubeda F, Koricheva J, LeRoy CJ et al (2009) From genes to ecosystems: a synthesis of the effects of plant genetic factors across levels of organization. Philos Trans R Soc Lond B Biol Sci 364 (1523): 1607–1616. Available: ISI:000265732200012. 10.1098/rstb.2008.0336 19414474 PMC2690499

[10] Tack AJM, Ovaskainen O, Pulkkinen P, Roslin T (2010) Spatial location dominates over host plant genotype in structuring an herbivore community. Ecology 91 (9): 2660–2672. 20957960 10.1890/09-1027.1

[11] Tack AJM, Johnson MTJ, Roslin T (2012) Sizing up community genetics: it’s a matter of scale. Oikos 121 (4): 481–488. Available: ISI:000301537200001.

[12] Wimp GM, Martinsen GD, Floate KD, Bangert RK, Whitham TG (2005) Plant genetic determinants of arthropod community structure and diversity. Evolution Int J Org Evolution 59 (1): 61–69. 15792227

[13] Bangert RK, Allan GJ, Turek RJ, Wimp GM, Meneses N et al (2006) From genes to geography: a genetic similarity rule for arthropod community structure at multiple geographic scales. Mol Ecol 15 (13): 4215–4228. Available: ISI:000241388800026. 17054514 10.1111/j.1365-294X.2006.03092.x

[14] Bangert RK, Lonsdorf EV, Wimp GM, Shuster SM, Fischer D et al (2008) Genetic structure of a foundation species: scaling community phenotypes from the individual to the region. Heredity 100 (2): 121–131. Available: ISI:000252585800004. 17047690 10.1038/sj.hdy.6800914

[15] Tack AJM, Roslin T (2011) The relative importance of host-plant genetic diversity in structuring the associated herbivore community. Ecology 92 (8): 1594–1604. 21905426 10.1890/10-2006.1

[16] Mattila A, Pakkanen A, Vakkari P, Raisio J (1994) Genetic Variation in English Oak (Quercus robur) in Finland. Silva Fennica 28 (4): 251–256.

[17] Vakkari P, Blom A, Rusanen M, Raisio J, Toivonen H (2006) Genetic variability of fragmented stands of pedunculate oak (Quercus robur) in Finland. Genetica 127 (1–3): 231–241. Available: ISI:000239164900020. 16850227 10.1007/s10709-005-4014-7

[18] Whitham TG, DiFazio SP, Schweitzer JA, Shuster SM, Allan GJ et al (2008) Perspective–Extending genomics to natural communities and ecosystems. Science 320 (5875): 492–495. Available: ISI:000255249300039. 10.1126/science.1153918 18436780

[19] Bacilieri R, Ducousso A, Petit RJ, Kremer A (1996) Mating system and asymmetric hybridization in a mixed stand of European oaks. Evolution Int J Org Evolution 50 (2): 900–908. Available: ISI:A1996UJ15600038. 10.1111/j.1558-5646.1996.tb03898.x 28568948

[20] Southwood TRE (1961) The Number of Species of Insect Associated with Various Trees. Journal of Animal Ecology 30 (1). Available: ISI:A1961WV72600001.

[21] Brandle M, Brandl R (2001) Species richness of insects and mites on trees: expanding Southwood. Journal of Animal Ecology 70 (3): 491–504. Available: ISI:000168978300010.

[22] Guichoux E, Lagache L, Wagner S, Leger P, Petit RJ (2011) Two highly validated multiplexes (12-plex and 8-plex) for species delimitation and parentage analysis in oaks (Quercus spp.). Molecular Ecology Resources 11 (3). Available: ISI:000289465500019. 10.1111/j.1755-0998.2011.02983.x 21481218

[23] Smouse PE, Peakall R (1999) Spatial autocorrelation analysis of individual multiallele and multilocus genetic structure. Heredity 82: 561–573. Available: ISI:000081096100012. 10383677 10.1038/sj.hdy.6885180

[24] Pritchard JK, Stephens M, Donnelly P (2001) Inference of population structure using multilocus genotype data. Genetics 155 (2). Available: ISI:000087475100039. 10835412 10.1093/genetics/155.2.945 PMC1461096

[25] Gossner MM, Chao A, Bailey RI, Prinzing A (2009) Native Fauna on Exotic Trees: Phylogenetic Conservatism and Geographic Contingency in Two Lineages of Phytophages on Two Lineages of Trees. American Naturalist 173 (5): 599–614. Available: ISI:000264812800006. 10.1086/597603 19296737

[26] Gossner MM (2004) Diversität und Struktur arborikolder Arthropodenzönosen fremdländischer und einheimischer Baumarten. Ein Beitrag zur Bewertung des Anbaus von Douglasie (Pseudotsuga menziesii (Mirb.) Franco) und Rotreiche (Quercus rura L.). Neobiota 5: 1–324.

[27] Wachmann E., Melber A., Deckert J., editor (2004) Wanzen. Keltern: Goecke & Evers

[28] Koch K, editor (1989) Die Käfer Mitteleuropas Krefeld: Goecke & Evers

[29] Böhme J, editor (2005) Die Käfer Mitteleuropas Band K-Katalog: Faunistische Übersicht.

[30] Simpson GG (1943) Mammals and the nature of' continents. American Journal of Science 241 (1): 1–31. Available: ISI:000200254500001.

[31] Koleff P, Gaston KJ, Lennon JJ (2003) Measuring beta diversity for presence-absence data. J Anim Ecology 72 (3): 367–382.

[32] R Core Team (2013) R: A language and environment for statistical computing.: R Foundation for Statistical Computing.

[33] Peakall R, Ruibal M, Lindenmayer DB (2001) Spatial autocorrelation analysis offers new insights into gene flow in the Australian bush rat, Rattus fuscipes. Evolution Int J Org Evolution 57 (5). Available: ISI:000183500900022. 10.1111/j.0014-3820.2003.tb00327.x 12836834

[34] Double MC, Peakall R, Beck NR, Cockburn A (2001) Dispersal, philopatry, and infidelity: Dissecting local genetic structure in superb fairy-wrens (Malurus cyaneus). Evolution Int J Org Evolution 59 (3). Available: ISI:000227943400014. 15856704

[35] Brandle M, Brandl R (2006) Is the composition of phytophagous insects and parasitic fungi among trees predictable. Oikos 113 (2): 296–304. Available: ISI:000237655400011.

[36] Smouse PE, Peakall R, Gonzales E (2008) A heterogeneity test for fine-scale genetic structure. Mol Ecol 17 (14): 3389–3400. Available: ISI:000257706500014. 18677808 10.1111/j.1365-294x.2008.03839.x

[37] Crutsinger GM, Cadotte MW, Sanders NJ (2009) Plant genetics shapes inquiline community structure across spatial scales. Ecology Letters 12 (4): 285–292. Available: ISI:000264067600002. 10.1111/j.1461-0248.2009.01288.x 19243408

[38] Crutsinger GM, Reynolds WN, Classen AT, Sanders NJ (2008) Disparate effects of plant genotypic diversity on foliage and litter arthropod communities. Oecologia 158 (1): 65–75. Available: ISI:000259819800007. 10.1007/s00442-008-1130-y 18766383

[39] Crutsinger GM, Strauss SY, Rudgers JA (2010) Genetic variation within a dominant shrub species determines plant species colonization in a coastal dune ecosystem. Ecology 91 (4): 1237–1243. Available: ISI:000277525300030. 20462137 10.1890/09-0613.1

[40] Crutsinger GM, Collins MD, Fordyce JA, Gompert Z, Nice CC et al (2006) Plant genotypic diversity predicts community structure and governs an ecosystem process. Science 313 (5789): 966–968. Available: ISI:000239817000043. 16917062 10.1126/science.1128326

[41] Crutsinger GM, Sanders NJ, Classen AT (2009) Comparing intra- and inter-specific effects on litter decomposition in an old-field ecosystem. Basic and Applied Ecology 10 (6): 535–543. Available: ISI:000270834400006.

[42] Dinnage R, Cadotte MW, Haddad NM, Crutsinger GM, Tilman D (2012) Diversity of plant evolutionary lineages promotes arthropod diversity. Ecology Letters 15 (11): 1308–1317. Available: ISI:000309395800012. 10.1111/j.1461-0248.2012.01854.x 22913753

[43] Haddad NM, Crutsinger GM, Gross K, Haarstad J, Knops JMH et al (2009) Plant species loss decreases arthropod diversity and shifts trophic structure. Ecology Letters 12 (10): 1029–1039. Available: ISI:000269742600003. 10.1111/j.1461-0248.2009.01356.x 19702636

[44] Johnson MTJ, Lajeunesse MJ, Agrawal AA (2006) Additive and interactive effects of plant genotypic diversity on arthropod communities and plant fitness. Ecology Letters 9 (1): 24–34. Available: ISI:000235306400005. 16958865 10.1111/j.1461-0248.2005.00833.x

[45] Robinson KM, Ingvarsson PK, Jansson S, Albrectsen BR (2012) Genetic variation in functional traits influences arthropod community composition in aspen (Populus tremula L.). PLoS ONE 7 (5): e37679 10.1371/journal.pone.0037679 22662190 PMC3360762

[46] Bernhardsson C, Robinson KM, Abreu IN, Jansson S, Albrectsen BR et al (2013) Geographic structure in metabolome and herbivore community co-occurs with genetic structure in plant defence genes. Ecol. Lett. 16 (6): 791–798. 10.1111/ele.12114 23601188

[47] Tack AJM, Gripenberg S, Roslin T (2012) Cross-kingdom interactions matter: fungal-mediated interactions structure an insect community on oak. Ecology Letters. 10.1111/j.1461-0248.2011.01724.x 22221681

[48] Legendre P, Borcard D, Peres-Neto PR (2005) Analyzing beta diversity: Partitioning the spatial variation of community composition data. Ecological Monographs 75 (4): 435–450. Available: ISI:000232649400001. 16544768

[49] Cottenie K (2005) Integrating environmental and spatial processes in ecological community dynamics. Ecology Letters 8 (11): 1175–1182. Available: ISI:000232535300006. 10.1111/j.1461-0248.2005.00820.x 21352441

[50] Soininen J, McDonald R, Hillebrand H (2007) The distance decay of similarity in ecological communities. Ecography 30 (1): 3–12. Available: ISI:000244419200001.

[51] Gossner MM, Getzin S, Lange M, Pašalić E, Türke M et al (2013) The importance of heterogeneity revisited from a multiscale and multitaxa approach. Biological Conservation 166: 212–220.

[52] Pimentel D (1961) Animal Population Regulation by the Genetic Feed-Back Mechanism. American Naturalist 95 (881): 65–79. Available: ISI:A1961WJ60100001.

[53] Greenstone MH (1984) Determinants of Web Spider Species-Diversity–Vegetation Structural Diversity Vs Prey Availability. Oecologia 62 (3): 299–304. Available: ISI:A1984SZ02700002. 28310881 10.1007/BF00384260

[54] Preszler RW, Boecklen WJ (1994) A 3-Trophic-Level Analysis of the Effects of Plant Hybridization on A Leaf-Mining Moth. Oecologia 100 (1–2): 66–73. Available: ISI:A1994PR64500008. 28307028 10.1007/BF00317131

[55] Fritz RS (1995) Direct and Indirect Effects of Plant Genetic-Variation on Enemy Impact. Ecological Entomology 20 (1): 18–26. Available: ISI:A1995QG17800003.

[56] Eisenbach J (1996) Three-trophic-level interactions in cattail hybrid zones. Oecologia 105 (2): 258–265. Available: ISI:A1996TV59300017. 28307091 10.1007/BF00328555

[57] Wimp GM, Whitham TG (2001) Biodiversity consequences of predation and host plant hybridization on an aphid-ant mutualism. Ecology 82 (2): 440–452. Available: ISI:000167064100012.

[58] Fritz RS, Price PW (1988) Genetic-Variation Among Plants and Insect Community Structure–Willows and Sawflies. Ecology 69 (3): 845–856. Available: ISI:A1988N720900032.

[59] Southwood TRE, Wint G, Kennedy EJ, Greenwood SR The composition of the arthropod fauna of the canopies of some species of oak (*Quercus*). Accessed 21 July 2014.

[60] Stone GN, Hernandez-Lopez A, Nicholls JA, Di Pierro E, Pujade-Villar J et al (2009) Extreme host plant conservatism during at least 20 million years of host plant pursuit by oak gallwasps. Evolution 63 (4): 854–869. 10.1111/j.1558-5646.2008.00604.x 19292826

[61] Bailey R, Schönrogge K, Cook JM, Melika G, Csóka G et al (2009) Host niches and defensive extended phenotypes structure parasitoid wasp communities. PLoS Biol. 7 (8): e1000179 10.1371/journal.pbio.1000179 19707266 PMC2719808

[62] Nekola JC, White PS (2001) The distance decay of similarity in biogeography and ecology. J Biogeography 26 (4). Available: ISI:000084684400013.

