# Supplementary figures and images for: Where Is the Extended Phenotype in the Wild? The Community Composition of Arthropods on Mature Oak Trees Does Not Depend on the Oak Genotype
Source: PLoS One. 2015 Jan 30;10(1):e0115733. doi: 10.1371/journal.pone.0115733 (PMC4321774; doi:10.1371/journal.pone.0115733)

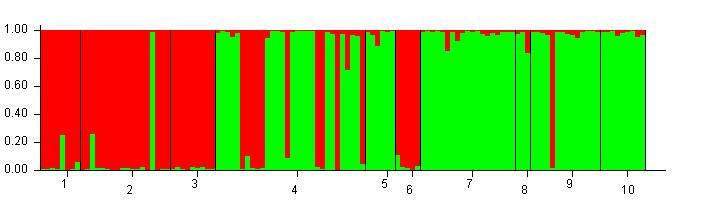

Supplement: S1 Fig — Each tree is represented by a vertical line divided in K colors. (JPG) [file pone.0115733.s001.jpg]
