# Supplementary material for: Where Is the Extended Phenotype in the Wild? The Community Composition of Arthropods on Mature Oak Trees Does Not Depend on the Oak Genotype
Source: PLoS One. 2015 Jan 30;10(1):e0115733. doi: 10.1371/journal.pone.0115733 (PMC4321774; doi:10.1371/journal.pone.0115733)
Supplement: S2 Table — Relationship between pair-wise community composition estimates (ln transformed Simpson dissimilarity index of true bugs and beetles) and oak genetic distances (OGD), spatial distance (Space), climate differences (Climate) and among the OGD, Space and Climate distance measures. The results of the Mantel test based on Pearson’s product-moment correlations are provided. Set1 indicates the first and Set2 indicates the second matrix of each Mantel test. (DOCX) [file pone.0115733.s003.docx]

**Table S2** **Mantel test between Oak genotype, space, climate and arthropod assemblages**

Relationship between pair-wise community composition estimates (ln transformed Simpson dissimilarity index of true bugs and beetles) and oak genetic distances (OGD), spatial distance (Space), climate differences (Climate) and among the OGD, Space and Climate distance measures. The results of the Mantel test based on Pearson’s product-moment correlations are provided. Set1 indicates the first and Set2 indicates the second matrix of each Mantel test.

| **Set1** | **Set2** | **r_M_** | **Significance** |
| --- | --- | --- | --- |
| **Beetles** |  |  |  |
| All beetles | OGD | 0.016 |  |
| Phytophagous leaf chewer | OGD | -0.0063 |  |
| Xylophagous saproxylics | OGD | 0.020 |  |
| Zoophagous saproxylics | OGD | 0.044 |  |
| Mycetophagous saproxylics | OGD | 0.0049 |  |
| All beetles | Space | 0.49 | ******* |
| Phytophagous leaf chewer | Space | 0.24 | ******* |
| Xylophagous saproxylics | Space | 0.12 | ******* |
| Zoophagous saproxylics | Space | 0.18 | ******* |
| Mycetophagous saproxylics | Space | 0.35 | ******* |
| All beetles | Climate PCA | 0.43 | ******* |
| Phytophagous leaf chewer | Climate PCA | 0.35 | ******* |
| Xylophagous saproxylics | Climate PCA | 0.13 | ******* |
| Zoophagous saproxylics | Climate PCA | 0.19 | ******* |
| Mycetophagous saproxylics | Climate PCA | 0.25 | ******* |
| **True bugs** |  |  |  |
| All true bugs | OGD | 0.020 |  |
| Phytophagous sucker | OGD | 0.021 |  |
| Zoophagous sucker | OGD | 0.044 |  |
| All true bugs | Space | 0.16 | *** |
| Phytophagous sucker | Space | 0.093 | *** |
| Zoophagous sucker | Space | 0.15 | *** |
| All true bugs | Climate PCA | 0.25 | *** |
| Phytophagous sucker | Climate PCA | 0.18 | *** |
| Zoophagous sucker | Climate PCA | 0.22 | *** |
|  |  |  |  |

The significance levels are as follows: *** p<0.001, ** p<0.01, and *p<0.05.
