# Supplementary material for: Where Is the Extended Phenotype in the Wild? The Community Composition of Arthropods on Mature Oak Trees Does Not Depend on the Oak Genotype
Source: PLoS One. 2015 Jan 30;10(1):e0115733. doi: 10.1371/journal.pone.0115733 (PMC4321774; doi:10.1371/journal.pone.0115733)
Supplement: S3 Table — Relationship between the pair-wise community composition estimates (ln transformed Simpson dissimilarity index of true bugs and beetles) and oak genetic distances (OGD), spatial distance (Space), climate differences (Climate) and among the OGD, Space and Climate distance measures. The results are the means of multiple Mantel tests based on Pearson’s product-moment correlations and are provided. Set1 indicates the first, Set2 indicates the second and ‘corrected for’ indicates the third matrix of each partial Mantel test. (DOCX) [file pone.0115733.s004.docx]

**Table S3** **Multiple Mantel test between Oak genotype, space, climate and arthropod assemblages**

Relationship between the pair-wise community composition estimates (ln transformed Simpson dissimilarity index of true bugs and beetles) and oak genetic distances (OGD), spatial distance (Space), climate differences (Climate) and among the OGD, Space and Climate distance measures. The results are the means of multiple Mantel tests based on Pearson’s product-moment correlations and are provided. Set1 indicates the first, Set2 indicates the second and ‘corrected for’ indicates the third matrix of each partial Mantel test.

| **Set1** | **Set2** | **Corrected for** | **r_M_** | **Significance** |
| --- | --- | --- | --- | --- |
| **Beetles** |  |  |  |  |
| All beetles | OGD | Climate PCA | -0.018 |  |
| Phytophagous leaf chewer | OGD | Climate PCA | -0.035 |  |
| Xylophagous saproxylics | OGD | Climate PCA | 0.0099 |  |
| Zoophagous saproxylics | OGD | Climate PCA | 0.031 |  |
| Mycetophagous saproxylics | OGD | Climate PCA | -0.017 |  |
|  |  |  |  |  |
| All Beetles | OGD | Space | -0.020 |  |
| Phytophagous leaf chewer | OGD | Space | -0.028 |  |
| Xylophagous saproxylics | OGD | Space | 0.0099 |  |
| Zoophagous saproxylics | OGD | Space | 0.030 |  |
| Mycetophagous saproxylics | OGD | Space | -0.032 |  |
|  |  |  |  |  |
| All beetles | Space | Climate PCA | 0.21 | *** |
| Phytophagous leaf chewer | Space | Climate PCA | 0.056 | * |
| Xylophagous saproxylics | Space | Climate PCA | 0.049 | * |
| Zoophagous saproxylics | Space | Climate PCA | 0.084 | ** |
| Myceophagous saproxylics | Space | Climate PCA | 0.26 | *** |
|  |  |  |  |  |
| All Beetles | Climate PCA | Space | 0.27 | *** |
| Phytophagous leaf chewer | Climate PCA | Space | 0.27 | *** |
| Xylophagous saproxylics | Climate PCA | Space | 0.083 | * |
| Zoophagous leaf chewer | Climate PCA | Space | 0.11 | ** |
| Mycetophagous saproxylics | Climate PCA | Space | 0.066 |  |
|  |  |  |  |  |
| **True Bugs** |  |  |  |  |
| True bugs | OGD | Climate PCA | 0.0019 |  |
| Phytophagous sucker | OGD | Climate PCA | 0.0079 |  |
| Zoophageous sucker | OGD | Climate PCA | 0.029 |  |
|  |  |  |  |  |
| True bugs | OGD | Space | 0.0067 |  |
| Phytophagous sucker | OGD | Space | 0.013 |  |
| Zoophageous sucker | OGD | Space | 0.034 |  |
|  |  |  |  |  |
| True bugs | Space | Climate PCA | 0.023 |  |
| Phytophagous sucker | Space | Climate PCA | -0.0096 |  |
| Zoophageous sucker | Space | Climate PCA | 0.033 |  |
|  |  |  |  |  |
| True bugs | Climate PCA | Space | 0.20 | *** |
| Phytophagous sucker | Climate PCA | Space | 0.15 | *** |
| Zoophageous sucker | Climate PCA | Space | 0.17 | ** |
|  |  |  |  |  |
